# Insecticide treated curtains and residual insecticide treatment to control *Aedes aegypti*: An acceptability study in Santiago de Cuba

**DOI:** 10.1371/journal.pntd.0006115

**Published:** 2018-01-02

**Authors:** Dennis Pérez, Patrick Van der Stuyft, María Eugenia Toledo, Enrique Ceballos, Francisco Fabré, Pierre Lefèvre

**Affiliations:** 1 Department of Epidemiology, Institute of Tropical Medicine Pedro Kourí, Havana, Cuba; 2 Department of Public Health, Institute of Tropical Medicine, Antwerp, Belgium; 3 Department of Public Health, Ghent University, Ghent, Belgium; 4 Department of Vector Control, Polyclinic Armando García, Santiago de Cuba, Cuba; 5 Department of Vector Control, Provincial Surveillance and Vector Control Unit, Santiago de Cuba, Cuba; Tulane University School of Public Health and Tropical Medicine, UNITED STATES

## Abstract

**Background:**

Within the context of a field trial conducted by the Cuban vector control program (*Aa*CP), we assessed acceptability of insecticide-treated curtains (ITCs) and residual insecticide treatment (RIT) with deltamethrin by the community. We also assessed the potential influence of interviewees’ risk perceptions for getting dengue and disease severity.

**Methodology/principal findings:**

We embedded a qualitative study using in-depth interviews in a cluster randomized trial (CRT) testing the effectiveness of ITCs and RIT in Santiago de Cuba. In-depth interviews (N = 38) were conducted four and twelve months after deployment of the tools with people who accepted the tools, who stopped using them and who did not accept the tools. Data analysis was deductive. Main reasons for accepting ITCs at the start of the trial were perceived efficacy and not being harmful to health. Constraints linked to manufacturer instructions were the main reason for not using ITCs. People stopped using the ITCs due to perceived allergy, toxicity and low efficacy. Few heads of households refused RIT despite the noting reasons for rejection, such as allergy, health hazard and toxicity. Positive opinions of the vector control program influenced acceptability of both tools. However, frequent insecticide fogging as part of routine *Aa*CP vector control actions diminished perceived efficacy of both tools and, therefore, acceptability. Fifty percent of interviewees did feel at risk for getting dengue and considered dengue a severe disease. However, this did not appear to influence acceptability of ITCs or RIT.

**Conclusion/significance:**

Acceptability of ITCs and RIT was linked to acceptability of *Aa*CP routine vector control activities. However, uptake and use were not always an indication of acceptability. Factors leading to acceptability may be best identified using qualitative methods, but more research is needed on the concept of acceptability and its measurement.

## Author summary

We aimed to understand what makes insecticide-treated curtains (ITCs) and residual insecticide treatment (RIT) with deltamethrin acceptable or not to users of these tools. In-depth interviews were conducted as part of a field trial conducted by the Cuban vector control program (*Aa*CP) to test the effectiveness of these tools in Santiago de Cuba. Perceived efficacy was the main reason for interviewees who accepted the tools. Constraints linked to manufacturer instructions were the main reason for not using the ITCs when offered at the start of the trial. People stopped using the ITCs due to perceived allergy, toxicity and low efficacy. Few heads of households refused RIT despite identifying various reasons for rejection, such as allergy, health hazard and toxicity. Positive opinions of the Cuban vector control program influenced acceptability of both tools. On the contrary, perceptions of dengue risk did not appear to influence acceptability of ITCs or RIT. Our findings add on the importance of the growing body of qualitative research assessing acceptability of health interventions.

## Introduction

Acceptability is the perception among individuals, organizations and entities involved in implementation that a given treatment, service, practice, or innovation is agreeable or satisfactory [1]. It is one of eight concepts identified in the literature for labeling and assessing outcomes of implementation processes. Implementation outcomes are preconditions to attain further intervention effectiveness [1, 2]. Acceptability studies of interventions or of specific tools used within an intervention (e.g., drugs, insecticides, vaccines, diagnostic procedures) are common in the health development field [3, 4]. Most of these studies tend to take the form of quantitative surveys and often measure acceptability through proxies such as uptake, short-term use, coverage and willingness to pay, among others.

Dengue is a vector-borne disease that is mainly transmitted by female *Aedes aegypti* mosquitos [5]. A mild episode of the disease can evolve to a severe and fatal hemorrhagic illness [6]. The disease is of growing public health importance in tropical and subtropical areas [7]. There is no effective antiviral therapy for the disease and a vaccine is still under research [8–11]. At present, vector control remains the cornerstone of prevention and control efforts [5, 10, 12–14].

*Ae*. *aegypti* control methods include the application of chemical products, the use of biological agents and environmental management of mosquito breeding sites [15]. However, it has become increasingly difficult to effectively control the vector and to counter expansion of the disease using existing tools [15]. Thus, developing new vector control methods is high on the dengue research agenda [15]. Some new tools (e.g., insecticide-treated materials, biological agents and genetic methods) are already the subject of operational research [16–20]. Some studies have shown promising results of their efficacy on dengue vector densities and potentially on dengue transmission, but are site and uptake dependent [16, 21, 22].

In Cuba, a dengue outbreak in Santiago de Cuba in 1997 [23] signaled that the national *Aedes aegypti* Control Program (*Aa*CP) was facing difficulties in sustaining its earlier successes in controlling the vector. Since then, the *Aa*CP has investigated the utilization of new vector control methods to impact dengue transmission and prevent outbreaks [24].

We conducted a qualitative study to assess acceptability of insecticide-treated curtains (ITCs) and residual insecticide treatment (RIT) by the community for control of *Ae*. *aegypti* and dengue. Our study was part of a field test of the tools conducted by the *Aa*CP in Santiago de Cuba. We also explored acceptability of the *Aa*CP and its routine vector control activities, and risk perceptions of getting dengue and severity of the disease to assess their potential influence on interviewee acceptability of ITCs and RIT.

## Methods

### The setting

Santiago de Cuba is the second largest city in Cuba and the capital of the province of the same name. It is located in the south-east area of the island and has 513,784 inhabitants [25]. *Ae*. *aegypti* proliferation is favored by the presence of, on average, four water-storage containers per household [26], high population density, uncontrolled urbanization, deficient solid and liquid waste management, high temperatures (28–34°C), and significant rainfall (1037.9 mm annually), among others. Despite ongoing vector control activities, infestation by *Ae*. *aegypti* persists, with an average house index for Santiago de Cuba of 2%, which can be substantially higher at the block level, leading to sporadic dengue outbreaks since 1997 [27–29].

A cluster-randomized controlled trial (CRT) was conducted in Santiago de Cuba from March 2011 to October 2012 to evaluate the effectiveness of ITCs and RIT to control *Ae*. *aegypti* [30]. The tools were distributed or applied by *Aa*CP field workers. During the trial, *Aa*CP routine vector control activities continued in both intervention and control clusters. Routine vector control activities included entomological surveillance, source reduction through periodic inspection of houses, larviciding (with temephos) of water-holding containers, selective adulticiding (fogging with cypermethrine and chlorpyrifos or perifocal residual spraying with deltamethrin) when *Aedes* breeding sites or dengue cases were detected, provision of health education information, promotion of community-based environmental management, and enforcement of mosquito control legislation [31]. Fines are applied to individuals who “obstruct enforcement of sanitary measures by the corresponding authority” (Legislative Decree 272, enacted February 20, 2001) by not allowing *Aa*CP field workers to enter the premises or do not permit spraying/fogging. Fines range from 100 to 300 Cuban pesos [32]; a significant financial impact on a household as in 2016, 300 Cuban pesos was nearly half the average monthly income [33].

### Study design

We conducted a small-scale qualitative study embedded [34] in the CRT conducted in Santiago de Cuba to test ICTs and RIT.

### The tools

**Fig 1:**
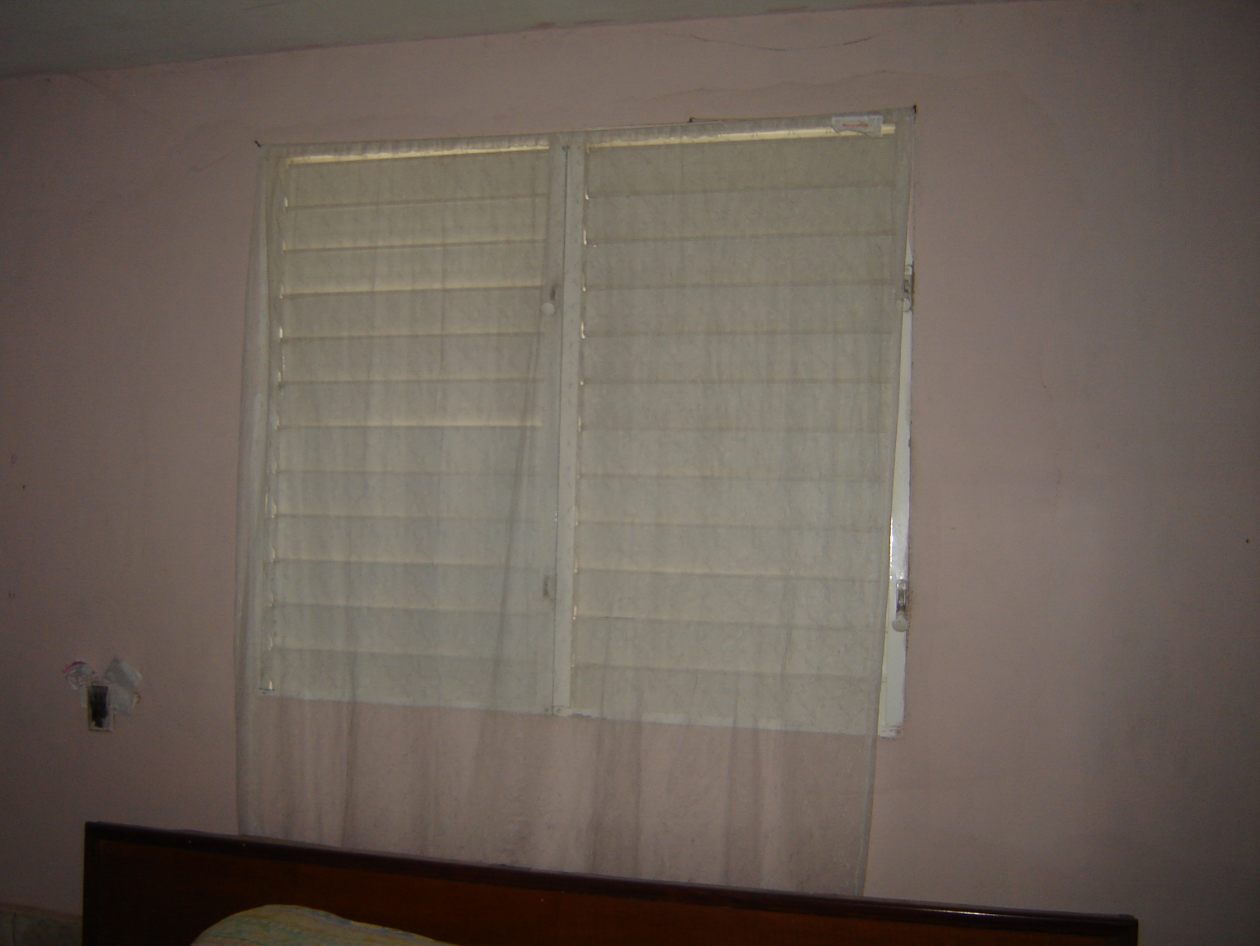
ITC hanging on a window.

**Fig 2:**
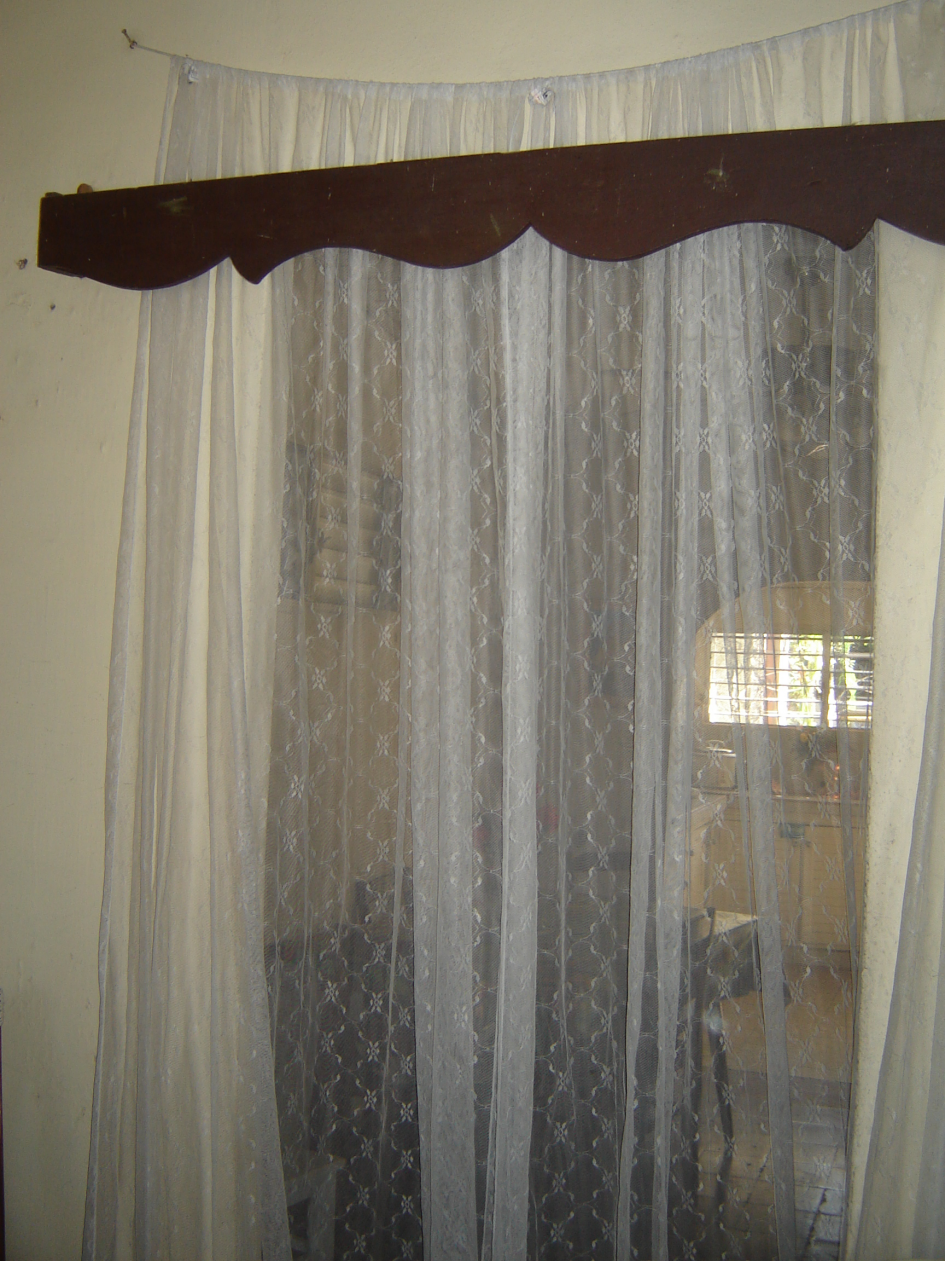
ITC hanging on an inside door.

ITCs were made from PermaNet polyester netting (Vestergaard-Frandsen, Switzerland) treated with a long-lasting formulation of deltamethrin (55mg/m^2^) and coated with a protectant (no details disclosed by the manufacturer) to prevent degradation of the insecticide when exposed to UV light. The ITCs retain their insecticidal properties and efficacy for about 2 years (information from manufacturer). The curtains were white patterned netting, 1.1m wide by 2.9m long. Placement of the ITCs took into consideration: *Ae*. *aegypti* resting behaviors; perceptions of the heads of households on the areas inside the house with greatest mosquito nuisance; and manufacturer instructions (i.e., ITCs should have little to no contact with sunshine; they should not be cut). Hanging places were negotiated with the head of household in order to increase acceptability. Heads of households preferred to hang the ITCs by windows or door openings, on closets or the wall (e.g., behind the bed), or attaching them to existing decorative curtains (Figs 1 and 2). A maximum of 3 ITCs were distributed per household; which was equal to the number of rooms in a standard house. In public spaces such as schools and offices, more curtains could be distributed.

Residual insecticide treatment (RIT) was conducted by spraying inside and outside the house every 4 months. K-Othrine 25 WG, supplied by Bayer Environmental Sciences Co. (25% deltamethrin formulation), was dissolved in water (20 grams/8 liters water) for a concentration of 25mg a.i./m^2^ sufficient to treat 200m^2^. While duration of the residual activity depends on the type of surface treated, it was expected to last 12 weeks on non-porous surfaces if not manipulated. The insecticide was sprayed on the outside of ground-level water tanks and the walls behind them, and on *Ae*. *aegypti* mosquito resting sites (e.g., under beds, inside and under closets) in the intra-domestic area. Before each application round, *Aa*CP workers were trained how to carry out the RIT activities, with support of a short video showing the correct application procedures. Supervisors conducted quality control visits during the application rounds.

### Data collection methods and selection of participants

In-depth interviews were used to explore the following three topics: 1) acceptability of the tools, such as appearance (in the case of the ITCs), disturbance (e.g., smell), perceived efficacy, effects on health, ecological and economic issues, and sources of information about the tools (e.g., formal, informal, rumors); 2) acceptability of the *Aa*CP, such as perceived efficacy, reputation, use of fines, house inspection (e.g., frequency, timing), routine control methods and their perceived efficacy; and 3) fear of dengue, risk perception of getting sick and of disease severity. Descriptive notes which included observations of interviewees’ facial expressions, tone of voice, and body language during the interview were taken and used for interpretation of the data [35].

The sampling unit was households in the intervention clusters where ITCs had been distributed and RIT rounds had been initiated. As part of the CRT, intervention clusters were randomized to receive only one of the two tools, thus individuals were interviewed about their experience with ITCs or RIT. Households were purposively selected from field trial records, and interviews were conducted four and twelve months after distribution (ITCs) or first application (RIT) of the tools. Initially the interview categories were households which had: 1) refused ITCs or RIT from trial onset, 2) accepted ITCs or RIT, 3) accepted ITCs with reluctance, or 4) stopped use of ITCs or RIT after one year (Table 1). As no information was available on households which accepted ITCs with reluctance, three user categories were created: 1) households which accepted the tools from trial onset, 2) households which accepted the tools and stopped using them after a period of time and 3) households which did not accept the tools at any time (YES, YES-NO and NO user categories, respectively) (Table 1). While a total of 20 interviews was initially planned, the total number increased during implementation as interviews were conducted until saturation, that is, no new information emerged from the data. Of the 41 households approached for an interview, 38 completed the interview, two refused to participate and one interviewee terminated their interview.

**Table 1:** Number of participants in in-depth interviews by user category. Santiago de Cuba, Cuba, 2011–2012.

User categories	Number of interviews (N = 38)	
	After 4 months	After 12 months
Refused ITC	4	—
Refused RIT	1	—
Accepted ITC reluctantly	0*	0*
Accepted ITC	6	1
Accepted RIT	11	1
Stopped using ITC	2	5
Refused RIT at least once after initial acceptance	3	4
Total	27	11

* No records of households belonging to this category

The interviews were conducted during two-week periods in September 2011 and April 2012. Interviews took place in a quiet place within the household, which allowed observing the context where the tools were deployed, verification of actual use of the ITCs and identification of use-related issues such as housing conditions. The interviews were conducted in Spanish by the principal investigator (DP), a sociologist with experience with qualitative inquiry. Interviews were conducted at random times in the morning, afternoon and early evening in order to reach individuals at home during the day as well as men and women who worked outside the home. The person who answered the door and was over 18 years of age was asked to participate in the interview. Those who did not wish to be interviewed were asked to identify another adult member of the household present at the time of the visit to be interviewed.

### Data analysis

All interviews were audio-taped after informed consent was obtained, and transcribed verbatim by DP and a professional transcriber. Data analysis was deductive, allowing for themes to emerge from the data. Data analysis was conducted with the support of QSR NVivo 9 software (QSR International Pty LTD, Melbourne, Australia). To increase internal validity, a sociologist (PL) not involved in data collection, reviewed the data and consistency of the coding system. Additionally, findings from the data analysis were discussed during the study period with the CRT research team, composed of professionals with diverse backgrounds such as epidemiology and vector control.

### Ethics

The research protocol was approved by the Research Ethics Committee of the Institute of Tropical Medicine Pedro Kourí, the Infectious Diseases Research Committee of the Cuban Ministry of Health and by the Institutional Review Board of the Institute of Tropical Medicine in Antwerp, Belgium. The Cuban National Vector Control Program (*Aa*CP) was provided with verbal and written explanations of the objectives and procedures of the study.

Obtaining written informed consent for the interviews was proposed in the initial protocol. However, during the first stage of data collection the written informed consent was replaced by oral informed consent due to fear and resistance of the interviewees to sign an official document. Participants were assured that their data would be confidential and only used for research purposes. Quotations are anonymous to protect participants' identity.

## Results

Information on the 38 interviewees is shown in Table 2. Age was not consistently collected; however, participants ranged in age from young adults to elderly individuals.

**Table 2:** Households participating in the study, by tool cluster, user category and sex of interviewee. Santiago de Cuba, Cuba, 2011–2012.

RESPONDENT CODE*	USER CATEGORY	SEX	RESPONDENT CODE*	USER CATEGORY	SEX
I-RIT 1	YES	F	I-ITC 1	YES	M
I-RIT 2	YES	F	I-ITC 2	YES-NO	F
I-RIT 3	YES	F	I-ITC 3	YES	F
I-RIT 4	YES	F	I-ITC 4	NO	M
I-RIT 5	YES-NO	M	I-ITC 5	YES	M
I-RIT 6	NO	F	I-ITC 6	NO	M
I-RIT 7	YES	M	I-ITC 7	NO	F
I-RIT 8	YES.NO	F	I-ITC 8	YES	F
I-RIT 9	YES-NO	M	I-ITC 9	YES-NO	F
I-RIT 10	YES	M	I-ITC 10	YES	F
I-RIT 11	YES	F	I-ITC 11	YES	M
I-RIT 12	YES	F	I-ITC 12	NO	F
I-RIT 13	YES	F	II-ITC 13	YES-NO	F
I-RIT 14	YES	M	II-ITC 14	YES-NO	F
I-RIT 15	YES	M	II-ITC 15	YES	F
II-RIT 16	YES-NO	F	II-ITC 16	YES-NO	F
II-RIT 17	YES	F	II-ITC 17	YES-NO	M
II-RIT 18	YES-NO	F	II-ITC 18	YES-NO	F
II-RIT 19	YES-NO	F			
II-RIT 20	YES-NO	F			

* Includes the tool and the data collection stage: I = September 2011 and II = April 2012.

### Acceptability of ITCs

None of the interviewees reported previous experiences with ITCs as the *Aa*CP had not previously used ITCs as a vector control method. Reasons for accepting ITCs were mentioned much less frequently after 12 months of use as compared with after 4 months. Interestingly, interviewees from households classified as NO users also noted reasons for use of the ITCs (Table 3).

**Table 3:** Reasons for acceptance and rejection of ITCs by user category, Santiago de Cuba, Cuba, 2011–2012.

	User Category		
	YES (n = 7)	YES-NO (n = 7)	NO (n = 4)
**REASONS TO ACCEPT**			
Perceived efficacy	6	2	1
Perceived as not harmful	6	1	1
Perceived as attractive	6	1	3
Do not generate inconvenience or annoyance	4	0	0
Beneficial	4	0	0
More advantages than disadvantages	3	0	0
To avoid nuisances	1	0	0
Permanent protection	0	0	1
Trust *Aa*CP who provided the ITCs	2	1	1
**REASONS TO REJECT**			
Constraints related to manufacturer instructions	0	0	4
Allergy	1	4	2
Low perceived efficacy	1	4	0
Toxicity	0	4	0
Insecticide is harmful to health	2	3	0
Issues not related to health	1	1	0
Smell	3	1	0
Skin reaction (rash)	2	0	0
Perceived as not attractive	0	1	0

Perceived efficacy, not being harmful and attractiveness were given as the main reasons to use ITCs, primarily by interviewees in the YES user category (n = 6):

“*The curtains are not ugly*, *they are beautiful*, *they have aesthetic… but the great utility they have is that they* [ITCs] *attract and kill insects*, *mainly mosquitos*. *I feel more secure…*” (female ITC YES user).

"*I think the curtain would be more effective*, *because it is permanent*, *because the Abate* [temephos], *at least in this area*, *has not been used for a while because there is no*… *with the* [indoor] *fogging when the smoke is gone it is finished*, *and if there were no mosquitos flying at that moment*, *it is not effective either*." (male ITC NO user).

Both women and men found attractiveness to be an added value for the ITCs, as shown in the following quotations:

“*Look*, *they* [ITCs] *are not ugly*, [they] *look like the ones I have here*. *So*, *when he* [*Aa*CP field worker] *came and he said they are like those ones*, *I said*: *perfect*.” (male ITC YES user).

“They [ITCs] *might eliminate the mosquito and serve you also in a way*, *let´s say decorative for the house…because they are even pretty*, *beautiful*, *it can be a way to decorate the house*.” (female ITC YES-N0 user).

“*This is another advantage*, *you put nice curtains*, *they kill the mosquito and we do not have to pay for them*.” (male ITC YES user).

However, the ITC did not always meet people´s preferences for the appearance and fabric quality of a curtain. A respondent who stopped using the curtains for aesthetic reasons stated:

“*I saw them* [ITCs] *as bed nets*, *I did not see quality in them… quality as curtains*. *…The material was a transparent and simple fabric*, *like for bed nets*.” (female ITC YES-NO user).

Constraints linked to manufacturer instructions on use of the ITCs (e.g., ITCs could not be washed until after six months of use, could not be cut and should have little to no contact with sunshine) was reported by all NO users as the reason for not accepting ITCs (Table 3):

“[the *Aa*CP field worker said] *that it was not compulsory to accept it* [the ITC]. *It was whether I wanted it or not*. *I told him I was not against using them but I did not have the right conditions* [according to ITC manufacturer instructions].” (male ITC NO user).

YES user interviewees also noted reasons for not using ITCs based on their experience, such as allergy, health hazard because of the insecticide, smell, and causing a local skin reaction (rash), among others. The main reasons for removing the ITCs reported by YES-NO users were allergy, toxicity and poor perceived efficacy (Table 3). Poor perceived efficacy of ITCs was primarily related to two issues: 1) lack of information on how the curtains work and 2) the combination of different control methods as part of the routine activities of the *Aa*CP.

The quantity and quality of information on the ITCs varied by the source of information. As reported by interviewees, information about the ITCs was provided either in community meetings prior to their distribution, by vector control field workers or by other users such as relatives and friends. However, regardless of source, interviewees did not have a clear idea of how the ITCs were supposed to work nor how to ascertain their effectiveness:

"*Those curtains… I thought that they would be inspected* [by *Aa*CP field workers], *they* [the ITCs] *would be useful*, *but if they are not inspected it is useless*, *because then what we know about them*, *we know nothing*." (female ITC YES-NO user).

“*For me they* [ITCs] *are not effective… We looked at the curtains to check if we could find mosquitos*, *but there was nothing*.” (female ITC YES user).

Some interviewees expected to avoid indoor spraying as an advantage of using the ITCs. That was not, however, the case as illustrated below:

“*The problem with this* [ITCs] *is that anyhow with the curtains they* [*Aa*CP field workers] *do* [indoor] *fogging*. *They come for spraying*. *We cannot say that with the curtains we have solved a problem… because they said with curtains there is no fogging because it was for testing the effect of the curtain*. *But you cannot reach any conclusion whether the curtains are favorable or unfavorable… Look*, *I would choose the curtain but without* [indoor] *fogging*.*”* (female ITC YES-NO user).

"*They were spraying* [in our house] *like in those that did not have them* [ITCs], *so*, *we need to revise such a measure*, *with curtain no spraying*, *but then if they do spraying to everyone including to those who have curtains*, *the curtains do not serve their purpose*, *do you understand me…*, *therefore you remove them* [the ITCs]." (female ITC YES-NO user).

### Acceptability of RIT

There were twelve, seven and one interviewees classified as YES, YES-NO and NO users, respectively. It was difficult to find interviewees who refused RIT at the start of the field trial (NO user category) in the trial records. Households classified as YES-NO users were reported in one out of four RIT applications, with the most frequent rejection of RIT being in the third round. Reasons for accepting RIT were mentioned much less frequently after 12 months than after 4 months of application. Perceived efficacy and no disturbances related to the insecticide were most frequently mentioned for accepting RIT, primarily by YES users (9 interviewees) (Table 4).

**Table 4:** Reasons for accepting or rejecting the application of residual insecticide treatment (RIT) by user category, Santiago de Cuba, Cuba, 2011–2012.

	User category		
	YES (n = 12)	YES-NO (n = 7)	NO (N = 1)
**REASONS TO ACCEPT RIT**			
Perceived efficacy	9	1	0
No disturbances related to the insecticide	9	1	0
Trust in *Aa*CP	6	0	0
Perception as not dangerous	3	0	0
Less frequent visits of *Aa*CP field workers	2	0	0
**REASONS TO REJECT RIT**			
Previous experiences with fogging/other residual treatment	3	2	0
Allergy	0	1	1
Smell	3	0	1
Toxicity	1	2	1
Poor perceived efficacy	1	2	0
Permanent contact with the insecticide	0	0	1
Expensive products	1	1	0
Health hazard	0	1	0
Environmental damage	0	1	0
Low perceived efficacy of fogging	0	0	1
Expansion of the insecticide when applied	1	0	0

Perceived efficacy was not only related to mosquitos, but to other insects as well:

“*This* [RIT] *is better*, *it kills more insects and it is not harmful for the children*. *Because the other* [fogging] *has a very strong smell*.” (female RIT YES user).

“*It* [the insecticide] *seems very good and it is strong*. *It has a very strong smell*, *a chemical smell*. *I think that is good to kill the mosquito*. *When they used it I felt there were no mosquitos*.*”* (female RIT YES user).

*“Well*, *you know*, *it* [the fumigation] *has nothing to do directly with the mosquito*, *but there are also other harmful insects*, *especially cockroaches… I saw it worked quite efficiently*.*”* (female RIT YES user).

The only NO user interviewee noted allergy, smell, toxicity, permanent contact with the insecticide, and poor perceived efficacy of fogging as reasons for rejecting RIT. Interviewees in the YES and YES-NO user categories also mentioned previous negative experiences with other residual treatment applications (Table 4). However, regardless of user category, interviewees revealed a lack of information on the specificities of RIT and confusion with previous experiences with different insecticides applied in a similar manner.

“*Because for most people*, *as they* [*Aa*CP field workers] *applied that white product that stain the walls…nobody wanted that product because they know how it is*.” (female RIT YES user).

“*They* [*Aa*CP field workers] *have been fumigating with smoke* [fogging], *also with a white liquid that stains the furniture… I have come and I have seen the stained furniture and my mother-in-law has told me that it is fumigation”* (female RIT YES user).

“*Here in the house there is a person who has a serious allergic condition*… *The last time that person was in contact with that white liquid*, *she turned red and we had to run* [to the hospital] …*we were afraid she was having an anaphylactic shock*.*”* (female RIT NO user).

### Acceptability of the *Aa*CP and its routine control activities

In general, interviewees had a positive opinion of the *Aa*CP and accepted the application of all the routine control methods employed by the program. Independent of the specific routine activities considered, most of the interviewees mentioned “usefulness” as the main reason for accepting *Aa*CP interventions, followed by the professionalism of the program staff. Conversely, negative opinions towards *Aa*CP program staff together with household lack of compliance with vector control recommendations were noted as the main reasons for possible rejection of the *Aa*CP, especially by interviewees with RIT application and interviewees in the YES user category (Table 5).

**Table 5:** Reasons for accepting and rejecting routine *Aa*CP activities and control methods, by tool cluster and user category, Santiago de Cuba, Cuba, 2011–2012.

	Tool cluster		User category		
	ITC (n = 18)	RIT (n = 20)	YES (n = 19)	YES-NO (n = 14)	NO (n = 5)
**REASONS TO ACCEPT**					
Usefulness of *Aa*CP	16	15	16	10	4
Professionalism of *Aa*CP staff	9	9	10	6	2
Social pressure and/or coercion)	6	5	5	4	2
*Aa*CP does the control actions for me (paternalism)	4	3	3	2	2
High investment by the State	1	4	2	2	1
**REASONS TO REJECT**					
Poor opinion of *Aa*CP field workers	6	11	9	7	1
Household lack of compliance with vector control recommendations	7	14	12	6	2
Lack of privacy	3	9	5	5	2
Frequency and hours of the field workers	7	6	3	6	3
Use of fines	1	4	2	2	1
Population has other priorities	1	4	1	3	1
Lack of information on *Aa*CP methods	1	3	3	1	0
Poor perceived efficacy of program activities and methods	0	4	1	3	0
No integrated strategies	0	2	1	1	0
Not feasible economically	0	1	0	1	0
Poor resolution to community problems by government	1	0	0	1	0

Other reasons why people might reject the *Aa*CP, such as the frequency and hours of field worker visits, lack of privacy and the use of fines, were linked to the *modus operandi* of the *Aa*CP. Although not numerous, interviewees held strong opinions:

“*In fact*, *in European countries*, *people cannot go inside of your house saying they are vector control program field workers unless you allow them to do it*. *That does not happen*. *Besides*, *they* [*Aa*CP field workers] *want to penalize you with a fine inside your own house*. *This goes against human rights*.” (male RIT YES-NO user).

“*If I do not allow them to come inside I get a fine*. *I do not want to get a fine*.” (female ITC YES-NO user).

Outdoor and indoor fogging with insecticides was strongly identified with the *Aa*CP and the presence of dengue cases or *Ae*. *aegypti* breeding sites in the area. The main reason for acceptance or rejection of fogging was perceived efficacy. However, environmental damage resulting from periodic intensified fogging campaigns was highlighted by half of the interviewees:

“*I see that birds disappear*, *and for example*, *here there are no insects*, *butterflies… It* [spraying] *is creating a brutal ecological imbalance here in Santiago de Cuba… there have been many years of spraying*.” (male RIT YES-NO user).

“*We have noticed that when there was smoke in the street* [outdoor space spraying] *it finished with the sparrows*, *with all animals that fly*, *even the vultures disappeared*.” (female RIT NO user).

Even those who gave reasons for rejecting indoor fogging accepted it, as it was perceived as mandatory. The following quotation reveals fears of the population to refuse its application:

“*Now there are many people who allow fumigating because they are threatened with a fine*, *I mean under pressure*.” (female ITC YES-NO user).

### Risk perception of dengue and disease severity

Interviewees who did not mention whether they personally felt at risk for dengue or whether dengue is a serious disease were asked specifically about these two issues. A majority of interviewees stated they perceived a risk for getting dengue and considered dengue to be a severe disease. However, this does not appear to have influenced acceptability of ITC or RIT (Table 6).

**Table 6:** Risk perception of dengue and perception of disease severity by user category, Santiago de Cuba, Cuba 2011–2012.

Perception of dengue		User category		
		YES (n = 19)	YES-NO (n = 14)	NO (n = 5)
Risk perception	Yes	13	7	3
	No	5	3	2
	n/r	1	4	0
Perception of severity	Yes	12	7	4
	No	4	3	1
	n/r	3	4	0

n/r = no response

In some cases, risk perception was linked to personal and familial experiences with the disease and extra concern for the health of small children:

“*I got dengue hemorrhagic fever*. *I was hospitalized*. *At that time almost everybody around here* [in the neighborhood] *got dengue*. *A high percentage of the people got it… I do not know any person with dengue around here now*, *but I think it is a very serious disease*.” (male RIT YES-NO user).

“*There are many diseases and my daughter is 6 years old*. *I have to take care of her… I have to allow them* [*Aa*CP field workers] *to fumigate*… *At least me*, *I am always careful about if there is a child or adult*, *and even myself*, *with fever to go quickly to the family doctor to get the test* [IgM] *to know if it is dengue or not*.” (female RIT YES user)

Some YES-NO and NO user category interviewees did not feel at risk of contracting dengue if they refused to use the ITCs or allow RIT, stating that people who follow “hygiene” and “preventive practices” in their households have less chance to get infected. Others felt protected by the application of other vector control methods:

“*I do not know why we would have the risk to be sick with dengue without using the curtains if we have our water containers well protected… I think I would not get infected*.” (female ITC YES-NO user).

“*Anyone could get dengue but I do not think that we* [the family] *are at risk of getting sick*. *We clean and try to take care of the house… Where there is a lack of household cleanliness and organization people could become sick because the risk is everywhere*. *But we have less chance to get the disease*… *I do not think I am not protected without the curtains*. *Besides they fumigate with a lot of smoke*. *I even tell them* [*Aa*CP field workers] *where to fumigate at home*.” (female ITC NO user)

It is important to note that there was no awareness on part of the interviewees of the actual risk of dengue transmission as most (27 of 38) did not know or were not sure about the epidemiological situation in their neighborhood. Interviewees complained about the lack of official information on *Ae*. *aegypti* breeding sites identified by *Aa*CP field workers and the presence of dengue cases in their neighborhood or the municipality, as illustrated below:

“*People in the street say that there are dengue cases*, *but there is no official information coming from a physician or a specialist*.” (female ITC YES user)

“*I heard* [in the street] *that someone is hospitalized with dengue but I cannot be sure because I do not trust the source of information*.” (male ITC YES user)

## Discussion

This study used in-depth qualitative research methods which allowed for the collection of “thick”, detailed data on the factors underlying acceptability of insecticide-treated curtains (ITCs) and residual insecticide treatment (RIT). Despite the many reasons provided for actual or potential rejection of ITCs and RIT by the interviewees, it was difficult to identify heads of households who refused to apply the tools or who stopped using them 12 months after distribution (ITCs) or application (RIT). In the cluster randomized trial conducted in Santiago de Cuba, RIT coverage was on average 97.2% [30]. In a similar trial conducted in Guantánamo Province, coverage reported for ITCs was 98.4% [24, 36]. These high coverage levels could be a contextual issue linked to the fact that both tools were distributed through the Cuban routine vector control program (*Aa*CP). Our results indicate that the *Aa*CP was highly accepted by the community in the intervention clusters in Santiago de Cuba, thus acceptability of the tools cannot be analyzed independently of the acceptability of the program and its routine vector control methods. This is in line with what has been reported in the literature on the importance of intervention distribution channels as a potential confounding factor in the assessment of acceptance or rejection of innovative control tools [37].

The perception of heads of households on the characteristics of the tools played a role in their usage. However, studies have shown that the acceptance of innovative vector control tools is not only determined by their technical attributes [37, 38]. ITCs were seen as innovative and very different from the control methods that the interviewees were used to, and this favored uptake of ITCs at the start. However, these perceptions affected long-term use as misconceptions on how the ITCs work led to low perceived efficacy as householders expected to observe dead mosquitos surrounding the curtains. A study conducted in Guatemala reported that the greatest perceived benefit of ITCs was that users could clearly see dead mosquitos beneath the curtains [39]. Follow-up surveys at 9 and 12 months post-ITC distribution in Iquitos, Peru reported a high percentage of participants who found ITCs effective in reducing mosquitos [40]. Likewise, a study conducted on the use and acceptance of screens made with netting (long-lasting insecticidal nets, LLINs) fitted to doors or windows in Mexico, found that the most notable benefit reported by users was the reduction in the amount of mosquitos at home, linked to a perceived reduction in mosquito biting [41]. Indeed, perceived efficacy has been reported as a strong motivation to use insecticide-treated materials [37, 40, 42, 43].

Most of the referenced studies used curtains or screens made with long-lasting insecticidal netting only on doors or windows [39, 41, 44]. In Peru, locations for placing the ITCs were suggested to the research staff by heads of households [45]. Similarly, in our study hanging places were negotiated with each head of household, with walls considered to be the primary place to hang the ITC. This could be a reason why “limited ventilation during the day time” was not mentioned among reasons for ITC rejection, as reported by Rizzo and colleagues [39].

In Cuba, insecticides targeting adult *Ae*. *aegypti* mosquitos have been widely and frequently applied by *Aa*CP as a routine control activity for dengue prevention. From 1981 to 1986, malathion and fenthion were used for fogging and perifocal residual spraying, respectively. From 1986 to date, pyrethroids (cypermethrin and lambda-cyhalothrin) have been used when *Ae*. *aegypti* positive breeding sites or dengue cases are detected (e.g., Santiago de Cuba in 1997 and 2006, and Havana 2001–2002). Other products such as bendiocarb have also been occasionally used [46–48].

The continuous use of insecticides as a core *Aa*CP control method could be the reason why interviewees in the RIT intervention cluster considered it to be a routine *Aa*CP control program activity, and perceived use of the tool as compulsory. Although in Cuba RIT is indeed compulsory during dengue outbreaks, it is not routinely used during interepidemic periods. Thus, even though all but one of the 20 interviewees accepted RIT, we cannot ignore the fact that interviewees also provided many reasons for rejection of residual insecticide treatment in and around the household. Hence, acceptability of RIT may be influenced by factors such as enforcement of mosquito control legislation (i.e., fines) and social pressure in a setting where avoiding dengue transmission is a high priority for the government and health authorities. Therefore, we cannot determine whether RIT was acceptable or not to the community as a dengue prevention tool.

RIT has been strongly recommended and widely implemented for malaria control [49], and a majority of national malaria control programs in Africa still rely on RIT [50]. It has been considered effective conditional on population willingness to accept its application, with acceptance varying according to location [49]. In some settings, political and social factors also became important drivers for acceptance that overrode factors that could motivate non-acceptance, as seen in a study on indoor residual spraying against malaria conducted in Manhiça district, rural Mozambique [51].

From an epidemiological point of view, continuation of routine vector control program activities, including frequent insecticide fogging in both intervention and control clusters, might not bias the results of the CRT in terms of effectiveness. However, it could have diminished perceived efficacy of both tools and, thus, acceptability for some heads of households. Indeed, intensive fogging could have led to low insect abundance, in spite of the fact that the intervention clusters were areas with the highest *Ae*. *aegypti* infestation levels according to baseline entomological surveillance data. This could have implications for integrated vector management strategies advocated in the literature for long-term sustainable vector control [5, 15] as the population may not be aware of or understand the technical reasons (e.g., resource availability, multi-disease control, increased effectiveness) for which a combination of approaches, methods and tools are being used.

Rizzo and colleagues reported that the acceptance of insecticide-treated materials (i.e., ITCs and drum screens) was particularly high in families who had experienced dengue [39]. Unlike other Latin American countries [52], in Cuba there is greater awareness of the implications and severity of dengue. However, we did not find an association between risk perception of getting sick and perceived severity of disease with acceptability of the vector control tools. This could be due to study limitations discussed below (e.g., sample size) and the presence of confounding factors specific to Cuba. In Cuba, dengue is not considered to be endemic, the outbreaks that occur are rapidly controlled with few deaths [53, 54], and information, education and communication (IEC) activities on dengue and *Aedes aegypti* decrease during inter-epidemic periods [54]. As shown in the present study, interviewees’ knowledge of the entomological and epidemiological situation in their neighborhood was very low; and risk perception of getting sick and perceived severity of disease were mainly driven by prior personal experiences with dengue.

Over the last three years, Latin America has confronted several outbreaks of viral diseases transmitted by *Ae*. *aegypti*; in particular the emergence of chikungunya in 2014 and Zika in 2015 [55]. In the particular case of Zika, even if disease risk is primarily focused on pregnant women and women of reproductive age, the threat of Zika is perceived by the general population as greater than that of dengue [56]. The potential influence of perceived risk of Zika on the acceptability of ITCs and RIT could be an important topic for future research in Cuba.

It has been acknowledged in the literature that acceptability of health interventions is a dynamic process, that changes with time and users’ experience [1]. This points to the need, when conducting acceptability studies, to identify the array of factors that could play a role in acceptability and their evolution over time. For this reason, we included a YES-NO category of users in the study and explored acceptability after 4 and 12 months of use or application of the tools.

Many acceptability studies are “product biased”, that is, it is often assumed that because the proposed tools are “good” for the health of the population, the beneficiaries will also perceive them as such. Thus, the focus is on how the products can be modified, if necessary, to be more readily accepted. But, as shown in this study, acceptability varies depending upon socio-cultural context. Reasons for rejection of ITCs in Santiago de Cuba included community preferences and expectations of how an “ordinary curtain” should look. This is in line with preferences and cultural connotations of color for insecticide-treated materials reported in the literature [39, 40, 57]. Our study findings also highlight the importance of beneficiaries’ values in understanding acceptability, which is especially important when there is rejection of or a strong reluctance towards an intervention. We identified the emergence of ecological values in Cuban society as well as issues of privacy associated with rejection of the *Aa*CP routine vector control actions.

Limitations of the study include the purposeful sampling of households and low number of interviewees in some of the user categories; thus, the context of the study should be kept in mind when interpreting the findings. We were not able to compare acceptability of the tools between the two data collection stages or within the three categories of users because of the smaller numbers in some of the categories and stages. This could also have affected the findings in terms of the relationship between acceptability of the tools and risk perception of the disease and disease severity. While purposive sampling does not allow generalization of results in terms of acceptability of ITCs and RIT outside the study area, a strength of the study is that it provided a detailed, in-depth understanding of the array of factors influencing acceptability of ITCs and RIT in this particular context. We found that reasons for acceptance or rejection of the tools were not isolated nor mutually exclusive. Thus, it is important that research themes or categories not be limited to what is already known or assumed by the research team, but include opportunities to identify factors related to the perceptions of the various intervention stakeholders and the socio-cultural context where the interventions or tools are deployed. These factors and the relationships among them could be better identified and explored through the use of qualitative methods in studies conducted preferably before designing quantitative surveys.

Finally, the issue of the appropriateness of using proxy indicators for measuring acceptability is one that needs to be better addressed in the literature. Significant efforts are made, especially in cluster randomized trials (CRTs), to reach high coverage and uptake levels in order to demonstrate intervention efficacy. However, the common assumption that there is congruence between reasons for using/not using and acceptance/rejection of interventions is an over simplification. We found RIT YES users who did not seem to really accept the RIT applications as well as ITC NO users who could potentially find ITCs acceptable. In order to avoid mistaken conclusions on what makes a health intervention acceptable or unacceptable for the beneficiaries, more research is needed on the concept of acceptability and its measurement.
